# Divergence of stem biomechanics and hydraulics between *Bauhinia* lianas and trees

**DOI:** 10.1093/aobpla/plab016

**Published:** 2021-04-08

**Authors:** Yan Xiao, Yu Song, Fu-Chuan Wu, Shu-Bin Zhang, Jiao-Lin Zhang

**Affiliations:** 1 CAS Key Laboratory of Tropical Forest Ecology, Xishuangbanna Tropical Botanical Garden, Chinese Academy of Sciences, Mengla, Yunnan 666303, China; 2 University of Chinese Academy of Sciences, Beijing 100049, China; 3 Center for Integrative Conservation, Xishuangbanna Tropical Botanical Garden, Chinese Academy of Sciences, Mengla, Yunnan 666303, China; 4 Horticulture Department, Xishuangbanna Tropical Botanical Garden, Chinese Academy of Sciences, Mengla, Yunnan 666303, China; 5 Yuanjiang Savanna Ecosystem Research Station, Xishuangbanna Tropical Botanical Garden, Chinese Academy of Sciences, Yuanjiang, Yunnan 653300, China

**Keywords:** *Bauhinia*, liana, modulus of elasticity, modulus of rupture, wood density, xylem anatomy

## Abstract

Liana abundance and biomass are increasing in neotropical and Asian tropical seasonal forests over the past decades. Stem mechanical properties and hydraulic traits influence the growth and survival of plants, yet the differences in stem mechanical and hydraulic performance between congeneric lianas and trees remain poorly understood. Here, we measured 11 stem mechanical and hydraulic traits for 10 liana species and 10 tree species from *Bauhinia* grown in a tropical common garden. Our results showed that *Bauhinia* lianas possessed lower stem mechanical strength as indicated by both modulus of elasticity and modulus of rupture, and higher stem potential hydraulic conductivity than congeneric trees. Such divergence was mainly attributed to the differentiation in liana and tree life forms. Whether the phylogenetic effect was considered or not, mechanical strength was positively correlated with wood density, vessel conduit wall reinforcement and sapwood content across species. Results of principle component analysis showed that traits related to mechanical safety and hydraulic efficiency were loaded in the opposite direction, suggesting a trade-off between biomechanics and hydraulics. Our results provide evidence for obvious differentiation in mechanical demand and hydraulic efficiency between congeneric lianas and trees.

## Teaser

Liana abundance and biomass have increased in neotropical and Asian tropical seasonal forests over the past decades. Stem mechanical properties and hydraulic traits influence the growth and survival of plants, yet the differences in stem mechanical and hydraulic performance between congeneric lianas and trees remain poorly understood. Here, we measured 11 stem mechanical and hydraulic traits for 10 lianas and 10 trees from Bauhinia grown in a tropical common garden. Our results showed that a trade-off existed between stem biomechanics and hydraulics, which provide evidence for obvious differentiation in mechanical demand and hydraulic efficiency between congeneric lianas and trees.

## Introduction

Mechanical support and water transport are two major functions of plant stems (Pratt *et al.* 2007; Bittencourt *et al.* 2016). Plant stems need adequate mechanical support to withstand their crown mass and prevent breakage from wind storm and arboreal animals (Read and Stokes 2006; Santini *et al.* 2013). In addition, plant stems mechanically support water transport through xylem (Niklas 1992). These two functions across species are realized by a range of stem traits (Bittencourt *et al.* 2016; Zhang *et al.* 2019), reflecting conflicting structural requirements in stems (Brodersen 2016). Stem biomechanics can affect photosynthetic carbon gain due to their effects on leaf arrangement and orientation and eventually influence plant survival and reproductive performance (Gartner 1995; Alvarez-clare and kitajima 2007). Therefore, characterizing the differentiation in stem functions and their determination are critical for understanding functional diversity and life-history strategies among different plant groups (Pratt *et al.* 2007).

The mechanical properties of stems can be described by measuring their modulus of elasticity (MOE) and modulus of rupture (MOR) (Niklas 1992; Gere and Timoshenko 1999). Usually, the more flexible stems have a lower MOE, while stiffer stems that are more resistant to bending have a higher MOR (Niklas 1992; Pratt *et al*. 2007; Onoda *et al.* 2010). Stem mechanical strength is positively related to wood density (King *et al.* 2006; Pratt *et al.* 2007; Niklas and Spatz 2010; Onoda *et al.* 2010; Santini *et al.* 2013), which is also highly dependent on stem anatomical characteristics associated with xylem, phloem and fibre matrix (Jacobsen *et al.* 2005, 2, 2007; Preston *et al.* 2006; Santini *et al.* 2013). In angiosperm, thick fibre wall contributes to high wood density and increases mechanical strength (Preston *et al.* 2006). Vessel wall reinforcement not only increases drought tolerance but also enhances mechanical safety (Jacobsen *et al.* 2007). Furthermore, increased xylem vessel lumen area and fraction of pith result in decreased mechanical strength (Santini *et al.* 2013; Rosell *et al.* 2014). Bark (including phloem and cambium) is an important fraction of stems. The contribution of bark to resisting bending forces is age-dependent and different among species (Niklas 1999). Santini *et al.* (2013) found that the proportion of bark was loosely negatively correlated with mechanical strength. Onoda *et al.* (2010) found that the contribution of bark to stem MOE was small even most species had thick bark. This is probably because bark in young stems is soft, which comprises thin tissue with low lignin content (Santini *et al.* 2013). In older stems, however, bark can be thickened with high content of lignin, resulting in stronger mechanical support (Niklas 1999). In addition, stem mechanical performance and the associations with stem morphological and anatomical properties have found to be influenced by environments (Read and Stokes 2006; Onoda *et al.* 2010). For instance, species growing in low-rainfall sites had higher wood density than those in high-rainfall sites at a given MOE and MOR (Onoda *et al.* 2010). Together, stem biomechanics is associated with a large body of its morphological, anatomical properties and growth environments (Preston *et al.* 2006; Jacobsen *et al.* 2007; Onoda *et al.* 2010, Santini *et al.* 2013).

Stem hydraulic efficiency is generally well quantified according to the Hagen-Poiseuille equation, which is highly and positively associated with hydraulically weighted vessel diameter and vessel fraction in xylem (Wagner *et al.* 1998; Jacobsen *et al.* 2007; Fan *et al.* 2017). Small vessels are thought to be resistant to drought-induced embolism but possess low hydraulic conductivity (Tyree and Ewers 1991). In addition, hydraulic efficiency is often negatively related to wood density (Pratt *et al.* 2007; Hoeber *et al.* 2014), even though the effect of wood density on hydraulics is indirect or non-causal (Lachenbruch and McCulloh 2014). The trade-off between mechanical safety and hydraulic efficiency across and/or within species was proposed to be related to stem properties and wood anatomical traits. However, experimental studies testing mechanical and hydraulic trade-off are relatively limited (Wagner *et al.* 1998; Jacobsen *et al.* 2007; Fan *et al.* 2017; Zhang *et al.* 2019).

Lianas are non-self-supporting structural parasites (Schnitzer and Bongers 2002) and their richness and biomass have been indicated to increase in neotropical forests (Schnitzer 2018; Schnitzer and van der Heijden 2019). Schnitzer (2005) proposed that lianas have a seasonal growth advantage over co-occurring trees, which allows lianas to increase in abundance in seasonal tropical forests in central Panama. Liang *et al.* (2007) also found that the species and individuals of lianas increased obviously from 1998 to 2006 with increased disturbance in a tropical rainforest, Xishuangbanna, Yunnan Province, Southwest China. The comparison between lianas and trees has attracted wide attention (Schnitzer *et al.* 2015; De Guzman *et al.* 2021; Medina-Vega *et al.* 2021). Comparing to self-supporting trees, lianas generally have lower wood density due to less carbon investment in mechanical support (Schnitzer and Bongers 2002; Dias *et al.* 2019). Additionally, previous studies also found that lianas exhibit higher hydraulic efficiency, predawn leaf water potentials and photosynthetic rates than co-occurring trees in seasonal tropical forests (Chen *et al.* 2015; Smith-Martin *et al.* 2019; van der Sande *et al.* 2019) and in savanna ecosystems (Zhang *et al.* 2016). However, tropical canopy trees and lianas differed in mechanical traits but converged in hydraulics (Zhang *et al.* 2019). Therefore, it remains unclear whether mechanical and hydraulic properties differ between co-occurring lianas and trees and how stem properties could shape the mechanical and hydraulic relationships among these two contrasting life forms.

Here, we measured stem mechanical, morphological and anatomical traits in 10 liana species and 10 tree species from *Bauhinia* grown in a tropical garden, Southwest China. *Bauhinia* is one of the largest genera of Leguminosae, comprising about 300 species with life forms of trees, shrubs and lianas, pantropically distributed in the world (Meng *et al.* 2014), giving us an opportunity to test the differences in stem biomechanics and hydraulics between closely related lianas and trees. Because these plants were grown in a common garden with the same environment, the differences in stem biomechanics and hydraulics across species can be attributed to inherited adaptive responses of the plants (Zhang and Cao 2009). Specifically, we aim to answer the following questions:

(i) How much variation in stem biomechanics and hydraulics is observed within the *Bauhinia* lianas and trees? Since stem biomechanics is strongly affected by the demands of the climbing habit in liana species, lianas may reduce mechanical strength and increase hydraulic efficiency compared with self-supporting trees. We first hypothesize that the differentiation in life forms will explain the largest part of variation in biomechanical and hydraulic traits.
(ii) Which stem properties (i.e. wood density, anatomical traits) influence stem biomechanics and hydraulics? Higher wood density is associated with higher dry mass cost, which indeed enhances mechanical safety (King *et al.* 2006; Plavcová *et al*. 2019), but this comes at the cost of hydraulic efficiency (Fan *et al.* 2017; Schnitzer 2018). We, therefore, hypothesize that stem biomechanics and hydraulic efficiency would be tightly linked with stem morphological and anatomical properties. Specifically, we expect traits related to wood hardness were positively correlated with biomechanics, while traits related to hydraulic conductivity were negatively correlated with biomechanical strength. In addition, the effect of bark on mechanical stiffness varies among species (Niklas 1999). Lianas usually possess higher stem flexibility than trees, we, therefore, hypothesize that bark content is higher in lianas than in trees, resulting in a decreased mechanical strength in lianas.

## Methods

### Study site and plant material

This study was conducted at Xishuangbanna Tropical Botanical Garden, Chinese Academy of Sciences (21º41′N, 101º25′E, elevation 570 m a.s.l.), Yunnan Province, Southwest China. Under the influence of Indian Ocean monsoon, this region has two distinct seasons: a rainy season (May to October) and a dry season (November to next April). The mean annual temperature is 21.7 ℃, with the monthly mean temperature being 15.9 ℃ in the coldest December and 25.7 ℃ in the warmest June. The mean annual precipitation is 1560 mm, with more than 80 % occurring during the rainy season.

A total of 10 liana species and 10 tree species within the *Bauhinia* were selected in this study (Table 1). These plants were grown in the common garden at least 4 years and were periodically watered. Six individuals per species were selected and tagged. Samples were taken at predawn. Seven to nine terminal and sun-exposed branches with a diameter of 8−10 mm were collected from each sampled individual. Hydraulic conductivity of terminal twigs and branches could be more directly related to leaf stomatal conductance and deployment (Pickup *et al.* 2005; Zhang and Cao 2009), thus regulates photosynthetic carbon gain and determines ecological interactions such as competition with neighbours (Onoda *et al.* 2010; Fanwoua *et al.* 2014). In addition, xylem vulnerability of terminal branches can provide a good estimation for hydraulic safety margin, which is related to plant drought-induced mortality (Meinzer *et al.* 2009). We stored the cutting branches in sealed plastic bags with moist tissue paper inside. We put these bags inside an insulated container with ice bags inside, and then returned to the laboratory for our experiment. We first measured the mechanical properties of the branches, with remaining samples being used to measure wood density and anatomical traits. All samples were collected during April 2019. In total, 11 traits were measured in this study (Table 2).

**Table 1.** List of sampled 20 *Bauhinia* (Leguminosae) species, life forms, average height (length) and diameter. The length of lianas and height of trees were estimated by tapes. The diameter at breast height (1.3 m height) was measured for trees, and the diameter point of measurement on lianas was measured according to Gerwing *et al* (2006). The nomenclature of plants is referred to Flora of China (http://www.iplant.cn/foc) and Chinese Field Herbarium (http://www.cfh.ac.cn/).

Species	Life form	Height/Length (m)	Diameter (cm)
*B. bidentata*	Liana	9.0 ± 0.9	2.2 ± 0.1
*B. carcinophylla*	Liana	8.7 ± 0.3	1.5 ± 0.0
*B. championii*	Liana	4.9 ± 0.5	1.2 ± 0.2
*B. curtisii*	Liana	12.0 ± 0.4	1.4 ± 0.1
*B. glauca* subsp. *tenuiflora*	Liana	7.6 ± 0.2	1.7 ± 0.0
*B. strychnifolia*	Liana	9.3 ± 1.3	2.0 ± 0.2
*B. scandens* var. *horsfieldii*	Liana	20.8 ± 1.8	2.5 ± 0.2
*B. touranensis*	Liana	20.5 ± 1.9	2.7 ± 0.1
*B. wallichii*	Liana	21.0 ± 2.6	3.9 ± 0.2
*B. yunnanensis*	Liana	6.4 ± 0.2	0.9 ± 0.0
*B. acuminata*	Tree	2.2 ± 0.1	6.3 ± 0.1
*B. × blakeana*	Tree	6.4 ± 0.2	13.5 ± 03
*B. brachycarpa*	Tree	6.5 ± 0.3	6.6 ± 0.3
*B. galpinii*	Tree	2.2 ± 0.4	2.6 ± 0.1
*B. monandra*	Tree	4.9 ± 0.2	8.7 ± 0.3
*B. purpurea*	Tree	6.7 ± 0.4	14.1 ± 0.7
*B. racemosa*	Tree	5.8 ± 0.4	10.3 ± 0.7
*B. rufescens*	Tree	4.1 ± 0.3	5.9 ± 0.3
*B. tomentosa*	Tree	3.6 ± 0.3	6.3 ± 0.2
*B. variegata*	Tree	10.3 ± 0.7	26.1 ± 1.5

**Table 2.** Traits measured in this study.

Trait	Abbreviation	Unit
Modulus of elasticity	MOE	MPa
Modulus of rupture	MOR	MPa
Wood density	WD	g cm^−3^
Bark content	BC	%
Sapwood content	SC	%
Pith content	PC	%
Vessel fraction	VF	%
Vessel density	VD	no mm^−2^
Conduit wall reinforcement	(*t*/*b*)^2^	/
Hydraulically weighted vessel diameter	*D*_h_	μm
Potential hydraulic conductivity	*K*_p_	kg m MPa^−1^ s^−1^

### Determination of stem biomechanics

The MOE (MPa) and MOR (MPa) of stems indicate the ability of stems to resist bending and breaking. Stem MOE and MOR were measured by a three-point bending method with a universal testing machine (Model 3343; Instron Corporation, Norwood, MA, USA). The diameter to length ratio was set as 1:20 and the vertical force was applied at 25 mm min^−1^. MOE was calculated as


$$\begin{array}{l} MOE=\frac{FL^{3}}{48I\delta } \end{array}$$

where *F* refers to the linear elastic region of load (N) and δ refers to deflection (mm) (Gere and Timoshenko 1999), and MOR was calculated as


$$\begin{array}{l} MOR=\frac{F_{max}LR}{4I} \end{array}$$

where *F*_max_ is the maximum load (N) and *R* is the radius of the stem (m). In both equations for MOE and MOR, *L* is the support span length (m) and *I* is the second moment of area (m^4^), with *I* = π*R*^4^/4.

### Wood density

A 7-cm segment was cut from the stems after biomechanical measurements. We measured fresh volume (cm^−3^) of the stem segment using the water displacement method and then stem segment was oven-dried at 70 ℃ for 72 h and weighed (DW, g). Wood density (WD, g cm^−3^) was determined as the ratio of DW to fresh volume.

### Stem anatomical and hydraulic traits

For the measurement of xylem structural and hydraulic traits, cross-sections of 20 *Bauhinia* species were made and then imaged using a microscope (smart zoom 5, Carl Zeiss, Germany). We took at least three images of each of seven to nine sampled stems for each species. We calculated hydraulically weighted vessel diameter (*D*_h_, μm), vessel density (VD, no mm^−2^), vessel fraction (VF, %), potential hydraulic conductivity (*K*_p_, kg m MP_a_^−1^ s^−1^) for hydraulic properties and we also measured conduit wall reinforcement [(*t*/*b*)^2^], the proportion of pith content (PC, %), bark content (BC, %) and sapwood content (SC, %) for morphological characteristics. We used a razor blade to smooth the stem surface, and then the proportions of pith, bark and sapwood were calculated as their width divided by the total stem diameter (Santini *et al.* 2013). All the anatomical parameters of the stem were measured using ImageJ (http://rsbweb.nih.gov/ij/).

We calculated the mean *D*_h_ as (Sperry *et al.* 1998):


$$\begin{array}{l} D_{h}=\;\Sigma d^{5}/\;\Sigma d^{4} \end{array}$$

where *d* is the vessel diameter (μm). *D*_h_ was used in our analyses rather than *d* because *D*_h_ is more directly related to xylem water transport. The conduit wall reinforcement was determined as (*t*/*b*)^2^, where *t* is the double cell wall thickness and *b* is the vessel diameter (Hacke and Sperry 2001). The VF was estimated as the ratio of total vessel lumen area to xylem area. *K*_p_ was calculated according to the Hagen–Poiseuille law (Poorter *et al.* 2010):


$$\begin{array}{l} K_{p}=\;\left(\frac{\pi \rho_{w}}{128\eta }\right)\;\times \;VD\;\times {D_{h}}^{4} \end{array}$$

where *η* is the viscosity of water (1.002 × 10^−3^ P_a_ s) and *ρ* is the density of water (998.2 kg m^−3^) at 20 ℃.

### Construction of phylogenetic tree

Young leaves of 20 *Bauhinia* were collected from the common garden and dried by chromotropic silica gel. After that, the DNA sequencing was performed by Personal Biotechnology (Shanghai, China). We assembled nuclear genome by GetOrganelle toolkit (Jin *et al.* 2020), and multiple alignments were manually adjusted with BioEdit v.7.1.3.0. Phylogenetic relationships were reconstructed using a maximum likelihood method in the IQ-TREE v.1.6.7.1 (Nguyen *et al.* 2015), the nucleotide substitution model was calculated by the jModelTest 2.0 program, and the optimal model of ‘TR+F+I+G4’ was selected (Darriba *et al.* 2012). Four *Cercis* species (*C. occidentalis*, *C. chinensis*, *C. glabra* and *C. chingii*) were chosen as the out-group because of their close relationship to *Bauhinia* (Meng *et al.* 2014). The ITS sequences (5.8s and 18s ribosomal RNA gene) of *Cercis* were downloaded from GenBank (http://www.ncbi.nlm.nih.gov). The phylogenetic relationships of the *Bauhinia* species in this study were shown **[see****Supporting Information—Fig. S1****]**.

### Data analysis

To improve the normal distribution and homogeneity of variance, all the values were log_10_-transformed before analysis. To assess trait variability, the quartile coefficient of dispersion was calculated as the formula of (Q3 − Q1) / (Q3 + Q1) × 100 %, where Q1 is the first quartile and Q3 is the third quartile. We used linear mixed-effects model to test for the distribution of variability for each trait. Life forms (lianas versus trees), species and individuals were introduced as nested random factors to assess how trait variability was distributed among three levels. Mixed-effects model was performed using the ‘lme’ function of ‘nlme’ package and the ‘varcomp’ function of ‘ape’ package. To assess trait differences between two life forms, we performed *t*-test with the ‘t.test’ function of ‘stats’ package. We compared the differences in relationships of mechanical properties with hydraulic traits between lianas and trees using a standardized major axis (SMA) with the ‘sma’ function of ‘smatr’ package.

To evaluate whether functional traits measured in this study were influenced by phylogenetic relationship, we separately calculated the phylogenetic signal for each trait by using Blomberg’s *K* statistics (Blomberg *et al.* 2003). The values of *K* were calculated using the ‘phylosignal’ function of ‘picante’ package. Values of *K* can be used to assess phylogenetic conservation. *K-*values >1 and <1 imply that close relatives are more similar and less similar, respectively, than expected under a Brownian motion model of trait evolution. *K*=1 implies that a trait is consistent with a Brownian motion model, while *K* = 0 shows that the trait has no phylogenetic signal (Blomberg *et al.* 2003).

Phylogenetically independent contrasts (PIC) were used to test for evidence of correlated evolution in stem traits by employing the ‘pic’ function of ‘ape’ package. We assessed relationships among 11 traits across 20 *Bauhinia* species with the ‘corr.test’ function of ‘psych’ package. A principal component analysis (PCA) was used to examine relationships among traits simultaneously with the ‘PCA’ function of the ‘FactoMineR’ package. Stepwise multiple regression analysis was used to test which traits contribute mostly to the MOE, MOR and *K*_p_ with the ‘lm’ function of ‘stats’ package and the ‘stepAIC’ function of ‘MASS’ package. Prior to stepwise multiple regression analysis, raw trait data were standardized using the ‘scale’ function of ‘base’ package. All analyses were carried out in R v.4.0.2 (R Core Team 2020).

## Results

### Differences in traits between lianas and trees

**Figure 1.**
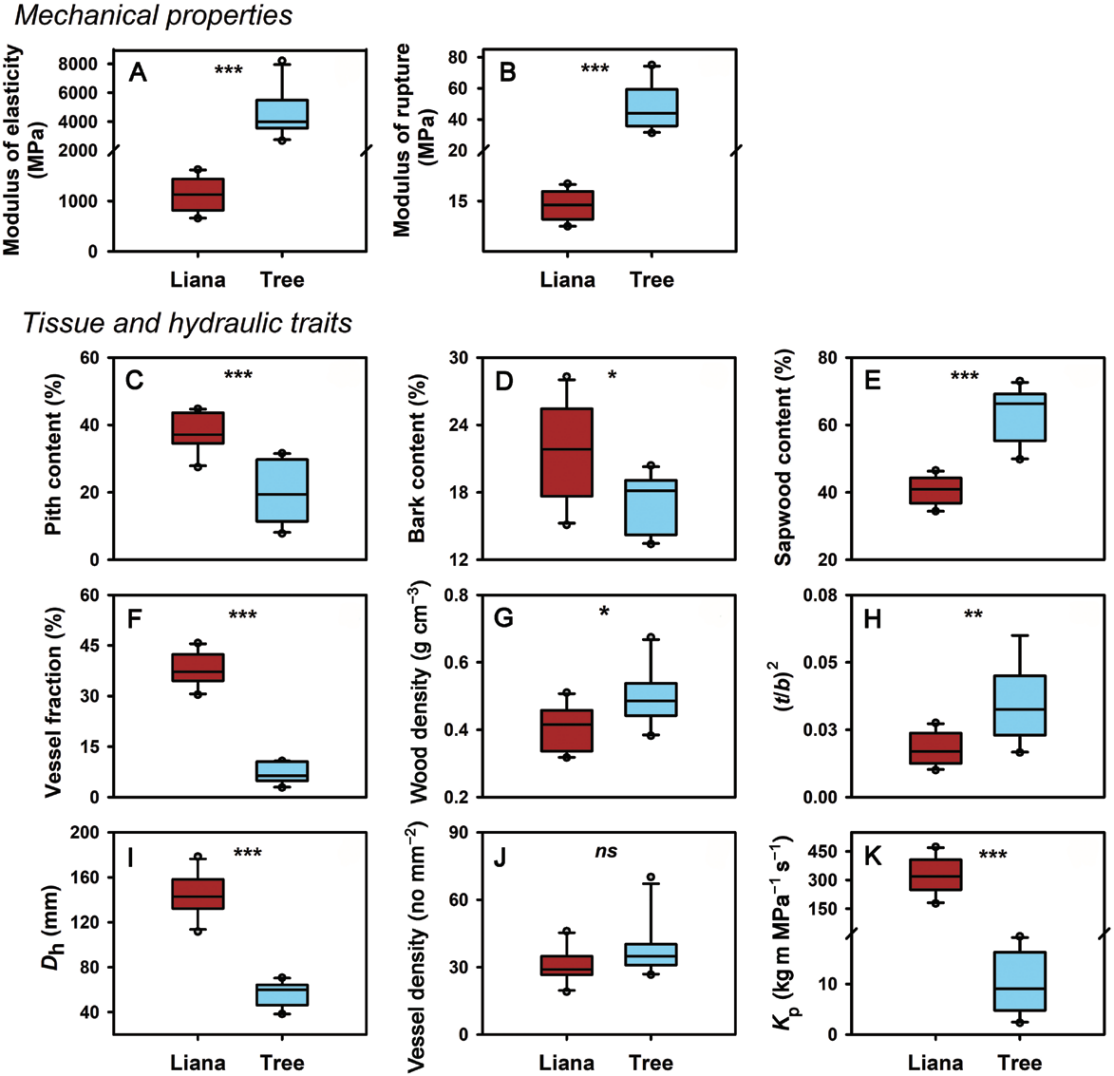
Differences in stem mechanics properties, tissue and hydraulic traits between lianas and trees. (*t*/*b*)^2^, conduit wall reinforcement; *D*_h_, hydraulically weighted vessel diameter; *K*_p_, potential hydraulic conductivity. Data were analysed using independent-samples *t*-test. ^ns^*P* > 0.05, *P < 0.05, ^**^P < 0.01, ^***^P < 0.001.

MOE and MOR were significantly higher in trees than in lianas (Fig. 1A and B). Lianas and trees significantly differed in stem cross-section fractions, with higher PC, BC and VF but lower SC in lianas than in trees (Fig. 1). In contrast, both WD and (*t*/*b*)^2^ were significantly higher in trees than in lianas (Fig. 1G and H). *D*_h_ and *K*_p_ were significantly higher in lianas than in trees, but there was no significant difference in VD between two life forms (Fig. 1I–K).

### Traits variation

**Figure 2.**
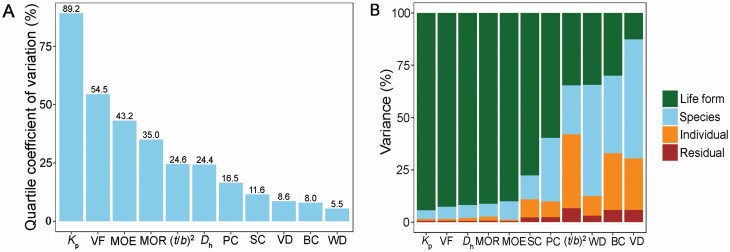
Quartile coefficient of dispersion (A) and variance partitioning of traits across life form, species, and individual (B). See Table 2 for trait abbreviations.

Among all traits, *K*_p_ showed the highest quartile coefficient of variance across species (89.2 %), followed by VF (54.5 %), MOE (43.2 %) and MOR (35.0 %). VD, BC and WD showed the least variation across species (5.5–8.6 %) (Fig. 2A). Most variation in *K*_p_, VF, *D*_h_, MOE, MOR, SC and PC was explained by life form, while variation in WD, BC and VD was mainly explained by species (Fig. 2B). Generally, individuals explained a small proportion of variation in all traits except for (*t*/*b*)^2^.

### Relationships between traits across species

To test whether variation in stem traits was shaped by phylogeny, we tested phylogenetic signals for 11 traits in *Bauhinia* species using the *K*-statistics **[see****Supporting Information—Table S1****]**. Except for PC and VD, all other traits showed a strong phylogenetic signal.

**Figure 3.**
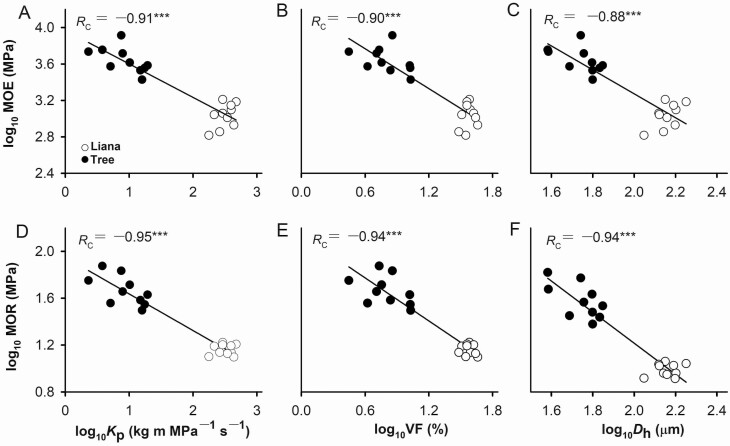
Relationships of modulus of elasticity (MOE) and modulus of rupture (MOR) with potential hydraulic conductivity (*K*_p_; A, D), vessel fraction (VF; B, E), and hydraulically vessel diameter (*D*_h_; C, F) across *Bauhinia* species. Pearson’s correlation coefficient (*R*_c_) was given. ^***^P < 0.001.

Because MOE and MOR were strongly correlated to each other, they showed similar patterns with other stem traits (Table 3). For instance, MOE and MOR were positively correlated with WD, SC and (*t*/*b*)^2^, but negatively correlated with BC, PC, *K*_p_, VF and *D*_h_ (Fig. 3; Table 3).

**Table 3.** Coefficients of Pearson’s correlation (above the diagonal) and phylogenetically independent contrast correlation (below the diagonal) between traits across species. Data were log_10_-transformed before analysis. See Table 2 for trait abbreviations. Significant values are indicated in bold. *P < 0.05, ^**^P < 0.01, ^***^P < 0.001.

	MOE	MOR	WD	BC	SC	PC	VF	VD	(*t*/*b*)^2^	*D*_h_	*K*_p_
**MOE**		**0.971** ^***^	**0.518** ^*^	**−0.501** ^*^	**0.879** ^***^	**−0.731** ^***^	**−0.904** ^***^	0.276	**0.621** ^**^	**−0.880** ^***^	**−0.909** ^***^
**MOR**	**0.859** ^***^		**0.635** ^**^	**−0.533** ^*^	**0.899** ^***^	**−0.777** ^***^	**−0.935** ^***^	0.411	**0.715** ^***^	**−0.938** ^***^	**−0.952** ^***^
**WD**	0.327	**0.564** ^**^		−0.158	**0.615** ^**^	**−0.693** ^***^	**−0.565** ^**^	**0.556** ^*^	**0.535** ^*^	**−0.651** ^**^	**−0.625** ^**^
**BC**	0.071	0.082	0.344		**−0.520** ^*^	0.183	**0.581** ^**^	−0.162	−0.383	**0.571** ^**^	**0.591** ^**^
**SC**	**0.602** ^**^	**0.558** ^*^	0.492*	0.003		**−0.899** ^***^	**−0.848** ^***^	0.355	**0.506** ^*^	**−0.857** ^***^	**−0.874** ^***^
**PC**	−0.402	**−0.517** ^*^	**−0.667** ^**^	−0.360	−0.831^***^		**0.676** ^**^	−0.434	**−0.488** ^*^	**0.724** ^***^	**0.720** ^***^
**VF**	**−0.546** ^*^	**−0.558** ^*^	0.002	0.239	−0.103	−0.181		−0.393	**−0.649** ^**^	**0.966** ^***^	**0.985** ^***^
**VD**	−0.238	0.031	0.268	0.144	0.082	−0.366	0.289		**0.526** ^*^	**−0.594** ^**^	**−0.500** ^*^
**(*t*/*b*)** ^**2**^	0.256	**0.483** ^*^	0.203	−0.121	−0.047	−0.079	−0.202	0.132		**−0.718** ^***^	**−0.697** ^***^
***D***_**h**_	−0.384	**−0.569** ^**^	−0.268	0.208	−0.286	0.208	**0.599** ^**^	−0.537^*^	−0.375		**0.994** ^***^
***K***_**p**_	**−0.559** ^*^	**−0.650** ^**^	−0.199	0.305	−0.304	0.085	**0.825** ^***^	−0.170	−0.379	**0.922** ^***^	

When phylogenetic effects were considered, both MOE and MOR were negatively correlated with VF and *K*_p_ (Table 3). However, the negative correlations between BC and mechanical traits were disappeared when phylogenetic effects were considered. Even the number of significant trait–trait relationships was reduced when phylogenetic effects were considered, most trait–trait relationships remained unchanged (Table 3). There were no significant differences in most SMA slopes and intercepts, but with significant shifts along a common slope between mechanical properties with hydraulic traits in lianas and trees (see Supporting Information—**Table S2**).

### Regression of MOE and MOR with stem traits

For MOE, 74 % variation was explained by WD and VF (Table 4). Although the contribution of WD was not significant in the multiple regression model of MOE, it increased the power of the model after entering. For MOR, 89 % variation was explained by WD, (*t*/*b*)^2^ and VF. In addition, 88 % of the variation in potential hydraulic conductivity was explained by vessel density and vessel fraction.

**Table 4.** Stepwise multiple regression models for modulus of elasticity (MOE), modulus of rupture (MOR) and potential hydraulic conductivity (*K*_p_) with wood and vessel traits in *Bauhinia* species. See Table 2 for trait abbreviations. ^**^P < 0.01, ^***^P < 0.001.

Equation	*R* ^2^	*P*-value
MOE = 3.44 × 10^−5^ + 0.284WD − 0.844VF^***^	0.74	0.000
MOR = 9.215 × 10^−5^ + 0.278WD^**^ + 0.320(*t*/*b*)^2**^ − 0.523VF^***^	0.89	0.000
*K*_p_ = −9.419 × 10^−5^ − 0.159VD + 0.884VF^***^	0.89	0.000

### Results of principle component analysis

**Figure 4.**
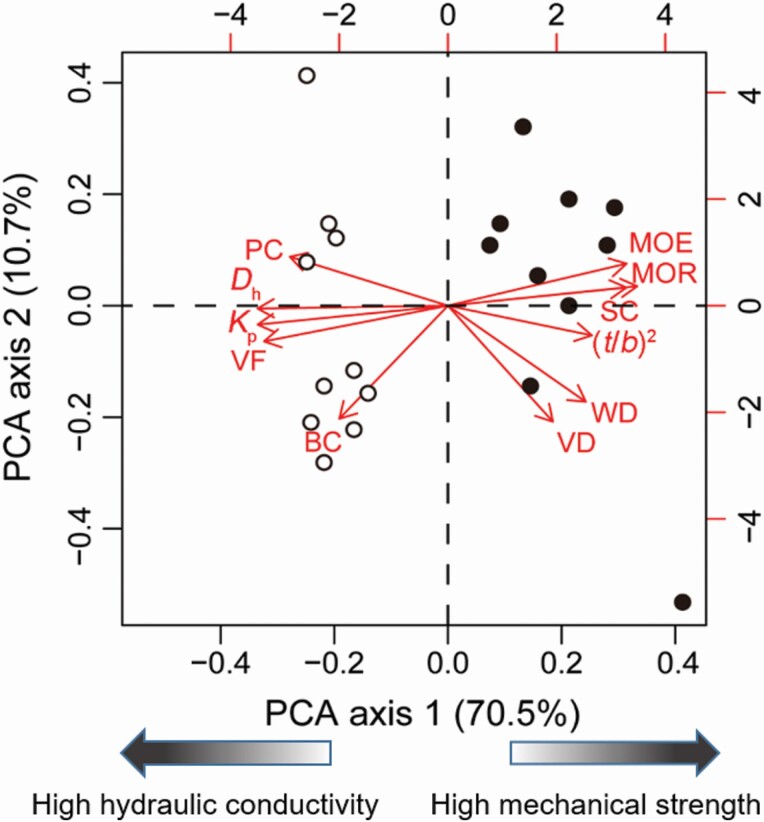
Positions of 11 stem traits, 10 lianas (open circle) and 10 trees (solid circle) on the first two axes of principal components analysis (PCA). Data were log_10_-transformed before analysis. See Table 2 for trait abbreviations.

The first PCA axis explained 70.5 % of the total variation in 11 traits (Fig. 4). Traits associated with mechanical strength (MOE and MOR) were clustered and loaded along the positive direction of PCA axis 1. Traits associated with hydraulic efficiency (*D*_h_, *K*_p_ and VF) were clustered and loaded along the negative direction of PCA axis 1. Therefore, the mechanical strength and hydraulic properties were loaded in the opposite direction along the first axis. The second axis only explained 10.7 % of the total variation. Lianas and trees were separated along the PCA axis 1, namely, liana species were mainly associated with the hydraulic efficiency traits while tree species were mostly associated with mechanical safety traits (Fig. 4).

## Discussion

In this study, we compared biomechanical and anatomical traits between *Bauhinia* liana and tree species (Fig. 1). We evaluated the effects of life form nested by species and individuals on the variation in biomechanical and hydraulic traits (Fig. 2). We found that life form explained the largest part of variation in biomechanical and hydraulic traits, supporting our first hypothesis. In addition, we analysed the linkages of stem biomechanics and hydraulic efficiency with stem morphological and anatomical properties from the perspective of phylogeny (Figs 3 and 4; Table 3).

### Divergence of stem biomechanics and hydraulics between congeneric lianas and trees

Studies on the differentiation in mechanical properties between lianas and trees are relatively rare. As observed in our study, there were significantly lower MOE and MOR in lianas compared with congeneric trees (Fig. 1A and B). Accordingly, we also observed significantly lower WD and (*t*/*b*)^2^ in lianas compared with congeneric trees (Fig. 1G and H), suggesting that lianas reduce carbon allocation to stem structural support compared with congeneric trees. Generally, lianas with lower mechanical strength possess more flexible stems, which are easier to bend or twist but more resistant to catastrophic fracturing than trees. In contrast, trees comprise homogeneous, stiff tissues that are more resistant to bending and twisting, but more susceptible to catastrophic fracture if the forces exceed the linear elastic limit of the stem (Schnitzer *et al.* 2015). The attachment and growth of lianas can affect the overall load of host plants (Schnitzer *et al.* 2015) and liana infestation of tree crowns can significantly increase tree mortality (Ingwell *et al.* 2010). Therefore, the mechanical differentiation between lianas and trees could represent different ecological strategies, which may provide a possible explanation for the increased abundance of lianas in neotropical and Asian tropical rainforests (Schnitzer 2005; Liang *et al.* 2007).

*Bauhinia* lianas exhibited a higher VF and *D*_h_ than congeneric trees (Fig. 1F and I), consequently, higher hydraulic conductivity in lianas than in trees (Fig. 1K). Plants invest more in conductive tissues (vessel lumen fraction) and thus increase hydraulic conductivity but decrease mechanical support (Lachenbruch and McCulloh 2014; Fan *et al.* 2017; Dias *et al.* 2019). The PCA results also showed that lianas and trees were separated at the side of hydraulic efficiency and the side of mechanical safety, respectively (Fig. 4). These results indicated a distinct divergence of stem biomechanics and hydraulics between lianas and trees. Furthermore, significantly higher proportion of BC was observed in lianas than in congeneric trees (Fig. 1D), indicating that higher phloem proportion points to efficient water use and photosynthate transport of lianas (Rosell *et al.* 2017). Unlike trees, lianas have relatively lower structural support, they, therefore, can allocate more resources to reproduction, canopy development and elongation of stems and roots (Schnitzer and Bongers 2002; Schnitzer 2018). This might be an evolutionary adaptation for non-self-supporting plants to achieve fast-growth strategies compared with self-supporting trees (Schnitzer *et al.* 2015).

### Associations of stem properties with its biomechanics and hydraulic efficiency

As we expected, our results showed that traits positively related to biomechanics were negatively related to hydraulic conductivity, supporting our second hypothesis. Previous studies have suggested that a greater conduit wall reinforcement as indicated by (*t*/*b*)^2^ can prevent conduit collapse under negative pressure (Hacke *et al.* 2001) and is highly related to greater vessel mechanical strength to keep hydraulic safety under water stress conditions (Jacobsen *et al.* 2007). With or without considering phylogenetic effects, (*t*/*b*)^2^ was significantly and positively associated with MOR across lianas and trees studied (Table 3), suggesting a correlated evolution between (*t*/*b*)^2^ and MOR. In addition, (*t*/*b*)^2^ had a positive effect on MOR (Table 4), suggesting that greater conduit wall reinforcement results in greater mechanical strength to against breaking. Previous studies showed that a greater degree of mechanical strength was associated with a higher cavitation resistance for stems (Jacobsen *et al.* 2005, 2007; Pratt *et al.* 2007). Although we did not measure the capacity of embolism resistance for the stems of sampled species, these results may also imply a covariation of cavitation resistance and mechanical strength in the stems within *Bauhinia* species.

Our results showed that sapwood content was tightly correlated with MOE and MOR among all species studied (Table 3), suggesting that increased sapwood content can significantly improve stem mechanical strength. After considering phylogeny, such strong positive associations were still existent, suggesting that sapwood was evolutionarily linked with mechanical strength in *Bauhinia* plants.

The bark is believed to provide mechanical support to the stem (Niklas 1999). As mentioned earlier, lianas had significantly higher bark content but significantly lower mechanical strength than trees (Fig. 1), which seems to support our third hypothesis that lianas with higher bark content could result in a decreased mechanical support. However, in this study, we found that the significant negative relationship between bark content and mechanical strength was disappeared when phylogenetic effects were considered (Table 3), suggesting that phylogeny has a significant effect on the association of bark content with biomechanics. Phylogenetic method should be used to test the relationships between bark content and biomechanical properties when taxa were closely related.

Wood density has shown to be positively correlated with stem strength and stiffness (van Gelder *et al.* 2006; Onoda *et al.* 2010), and thus can be used as an indicator of plant support cost (King *et al.* 2006). We indeed found that wood density had a positive effect on mechanical strength measured by MOE and MOR across *Bauhinia* species (Table 4). Xylem vessels are the pathways for long-distance transport of water and nutrients from roots to leaves (Ooeda *et al.* 2018). As Hagen-Poiseuille theory predicted (Tyree and Ewers 1991), we found that higher *K*_p_ was mainly attributable to higher *D*_h_ and VF in *Bauhinia* lianas and trees (Table 4). Wider vessel, which was tightly correlated with higher fraction of vessels in stems (Table 3), was a key anatomical feature for hydraulic conductivity of lianas (Anfodillo *et al.* 2012).

Our results showed that there were strong negative relationships of *K*_p_ with MOE and MOR across *Bauhinia* species examined, with or without considering phylogeny (Fig. 3; Table 3). This suggests an evolutionary trade-off between mechanical strength and hydraulic efficiency across *Bauhinia* liana and tree species. Such trade-off is mainly regulated by vessel size and fraction because higher VF and *D*_h_ point to higher *K*_p_ meantime at the cost of hydraulic safety and mechanical stability (Wagner *et al.* 1998; Hacke *et al.* 2006; Fan *et al.* 2017). This also can be confirmed by PCA results, in which stem traits related to mechanical strength and hydraulic efficiency were in the opposite directions (Fig. 4), consistent with previous studies (Pittermann *et al.* 2006, Fan *et al.* 2017). When large vessels in xylems are closely spaced, more resources are allocated to conductive tissues than to mechanical support (Wagner *et al.* 1998). Taken together, vessel fraction regulates stem biomechanics and hydraulics in *Bauhinia* lianas and trees.

## Conclusions

This study tested the differences in mechanical strength and hydraulic efficiency between congeneric lianas and trees, and we found that mechanical strength and hydraulic efficiency differed strikingly between two life forms. We also found that such differentiation in life form contributes to large proportion of variation in biomechanics and hydraulics in *Bauhinia* species. Our results provide a possible explanation for fast growth of lianas over congeneric trees. Findings from this study also have important implications for life-history strategies in non-self-supporting plants. Moving onwards, to further assess life-history strategies in non-self-supporting plants, more studies from different ecosystems, are needed.

## Supporting Information

The following additional information is available in the online version of this article—

**Table S1**. Phylogenetic signal for 11 traits.

**Table S2**. Test of the standardized major axis regression slopes, intercepts and shifts along the common slopes for relationships of mechanical properties with hydraulic traits between lianas and trees.

**Figure S1**. Phylogenetic tree for the 20 *Bauhinia* species examined using ITS sequence.

plab016_suppl_Supplementary_Materials

## Data Availability

Data and R-code used in this paper are available on Figshare https://figshare.com/s/73c4156bc53aae1db841.
