# Supplementary material for: Divergence of stem biomechanics and hydraulics between Bauhinia lianas and trees
Source: AoB Plants. 2021 Apr 8;13(3):plab016. doi: 10.1093/aobpla/plab016 (PMC8114228; doi:10.1093/aobpla/plab016)
Supplement: plab016_suppl_Supplementary_Materials [file plab016_suppl_supplementary_materials.pdf]

Table S1. Phylogenetic signal, quantified as Blomberg's *K*, for 11 traits across species.

Trait	*K*	*P*-value
Modulus of elasticity	1.889	**0.001**
Modulus of rupture	2.018	**0.001**
Wood density	0.604	**0.024**
Bark content	0.609	**0.036**
Sapwood content	1.092	**0.001**
Pith content	0.549	0.053
Vessel fraction	2.483	**0.001**
Vessel density	0.335	0.514
Conduit wall reinforcement	0.560	**0.036**
Hydraulically weighted vessel diameter	3.257	**0.001**
Potential hydraulic conductivity	3.867	**0.001**

Significant phylogenetic signals are shown in bold.

Table S2. Test of the standardized major axis regression slopes, intercepts, and shifts along the common slopes for relationships of mechanical properties with hydraulic traits between lianas and trees.

y ~ x	Common slope/slope			Intercept			Shifts along the common slope
	Lianas	Trees	*P*	Lianas	Trees	*P*	*P*
MOE ~ K_p	*0.65*		*0.08*	*4.66*	*4.25*	*0.08*	**<0.001**
MOE ~ VF	**2.40**	**-0.73**	**0.02**				
MOE ~ D_h	1.89		0.26	**-1.05**	**0.34**	**<0.001**	0.34
MOR ~ Kp	0.34	2.05	0.68	2.11	2.02	0.68	**<0.001**
MOR ~ VF	-0.84	-0.66	0.63	2.32	2.26	0.64	**<0.001**
MOR ~ D_h	0.83	-1.32	0.31	3.53	3.58	0.67	**<0.001**

If the slopes were not significantly different between liana and tree species, then the common slopes were given; if no significant differences in slopes and shifts in intercepts were detected, shifts along the common slopes were then tested for.

Data were log_{10}-transformed before analysis. Statistically significant results ($P < 0.05$) were highlighted in bold; statistically marginally significant results ($0.05 < P < 0.10$) were indicated with italics.

MOE, modulus of elasticity; MOR, modulus of rupture; K_p, potential hydraulic conductivity; VF, vessel fraction; D_h, hydraulically vessel diameter.

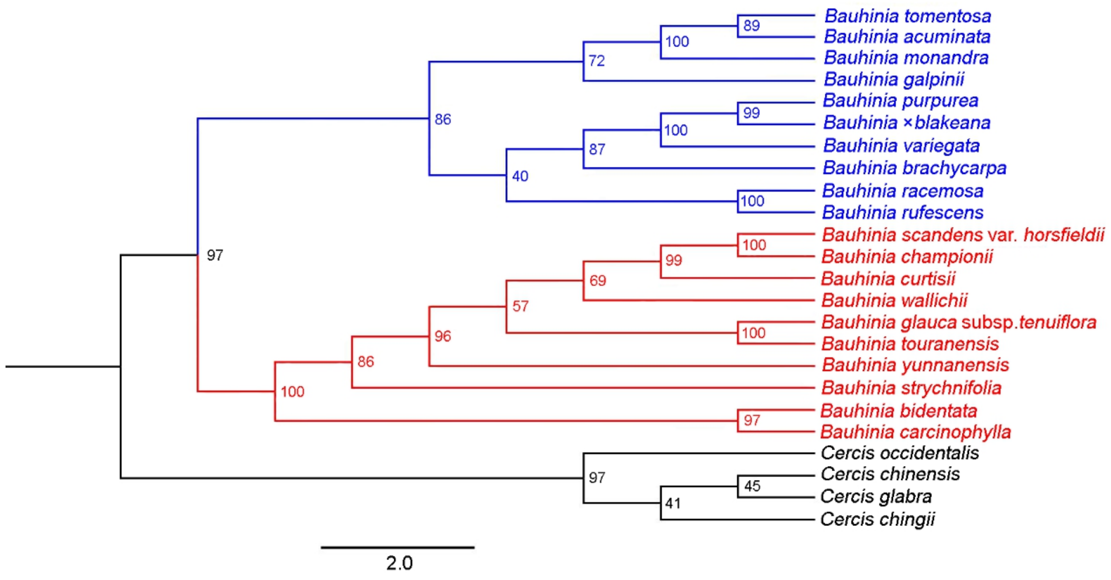

Figure S1. Phylogenetic tree of the 20 *Bauhinia* species examined, using ITS sequence. Red and blue colors represent lianas and trees, respectively. *Cercis* species were used as out-group.
